# Association of follicle-to-oocyte index and clinical pregnancy in IVF treatment: A retrospective study of 4,323 fresh embryo transfer cycles

**DOI:** 10.3389/fendo.2022.973544

**Published:** 2022-10-03

**Authors:** Peiyi Li, Zhiyun Chen

**Affiliations:** The Assisted Reproduction Center, Huizhou Central People’s Hospital, Huizhou, China

**Keywords:** embryo transfer, clinical pregnancy, *in vitro* fertilization, ovarian responsiveness, controlled ovarian hyperstimulation, follicle-to-oocyte index

## Abstract

**Objective:**

The aim of this study is to investigate whether the follicle-to-oocyte index [FOI: (number of retrieved oocytes/antral follicle count) × 100] was associated with clinical pregnancy after fresh cleavage transfer.

**Design:**

The framework used to organize this study is retrospective cohort analysis.

**Setting:**

The study was performed in a single *in vitro* fertilization center in a public hospital.

**Patients:**

In total, 4,323 fresh embryo transfer cycles from 1 August 2011 to 31 January 2022 were retrospectively analyzed. Data were designated into three groups according to FOI tertile values.

**Interventions:**

There are no interventions in this study.

**Main outcome measure:**

The primary outcome measure is the clinical pregnancy rate (CPR).

**Results:**

A total of 4,323 patients were included in the study. According to their FOI, patients were divided into low (FOI ≤ 0.70, n = 1,434), medium (FOI = 0.71–0.95, n = 1,070), and high (FOI = 0.96–1.00, n = 1,819) tertile groups. A significant statistical increase in the CPR from the lowest to the highest tertile FOI group was detected (47.28%, 51.78%, and 51.57%; *P* =0.026). After adjusted for potential confounders, multivariate logistic regression analysis revealed a positive association between FOI and CPR [odds ratio (OR) = 1.57; 95% confidence interval (CI): 1.18–2.11]. Each standard deviation increments in FOI (SD = 0.24) corresponded to a 20% increase in the CPR. Trend analysis also showed that FOI tertile groups were positively associated with CPR (*P* for trend = 0.010). Smooth curve fitting indicated the existence of a linear relationship across the entire range of FOI. No optimal cutoff value of FOI for prognosing CPR was found in smooth curve fitting analysis. Moreover, subgroup analyses suggested that the association was significantly stronger in the single cleavage transfer cycle (OR = 2.04; 95% CI: 1.14–3.65).

**Conclusions:**

FOI is an independent variable in prediction for CPR in fresh embryo transfer cycle, especially in the single cleavage transfer cycle.

## Introduction

Controlled ovarian hyperstimulation (COH) is the one of the pivotal processes during *in vitro* fertilization (IVF) treatment, enabling the development of multiple oocytes and best embryo selection for transfer (1). Follicle-stimulating hormone (FSH) stimulates follicle development (2), and the adequate reactivity of antral follicles to FSH is a sign of normal reproductive functions (3). An appropriate ovarian response to COH is a prerequisite for achieving successful pregnancy, and different ovarian responses would affect IVF outcomes (4).

Several ovarian biomarkers, including the basal FSH level, anti-Müllerian hormone (AMH) level, and antral follicle count (AFC), were used to predict ovarian response, but they had certain limitations in practice (5, 6). Hence, the capacity of the aforementioned markers to predict IVF outcomes remained disputable (6, 7).

Consequently, there is an increasing need for effective ovarian response tools for the prediction of successful IVF pregnancy. In recent years, more complex and quantitative biomarkers for predicting ovarian function had been investigated, such as follicular output rate (FORT) (8, 9) and ovarian sensitivity index (OSI) (10, 11). FORT represents the ratio of the number of preovulatory follicles divided by the AFC, whereas OSI was calculated by dividing the number of retrieval oocytes by the total FSH dosage administrated in COH. Both higher OSI (12, 13) and higher FORT (14, 15) were reported to associate with improved pregnancy outcomes in IVF treatment. However, because of their definitions, these markers had some limitations. OSI does not differentiate the type of GnRH analog, the type of gonadotropin, the starting dose, or the gonadotropin regimen. FORT does not consider the actual number of oocytes obtained, which was the effective result of the COH process and found to be more relevant to live birth in IVF treatment (16). Hence, both the number of oocytes retrieved and AFC should be considered as quantitative aspects of ovarian responsiveness.

In 2018, follicle-to-oocyte index (FOI) was proposed as a new parameter to address ovarian responsiveness (17). As FOI represents the ratio of the number of retrieved oocytes and AFC before COH, FOI might best grasp the developmental dynamics of follicles upon FSH stimulation (17). Furthermore, FOI is also an alternative approach to identify the hypo-response, which was first introduced 10 years ago to refer to women with an impaired ovarian response to exogenous gonadotropin stimulation. For instance, a woman who had an AFC of 14 and retrieved oocytes of 8 might still implied hypo-response upon COH, although these values were well above the threshold of poor response to ovarian stimulation (18). In this physiological context, we propose a hypothesis that women with normal sensitivity of antral follicles to circulating FSH have higher chances of achieving pregnancy success. FOI higher than 50% was assumed to represent normal ovarian responsiveness (17). So far, the association between FOI and IVF pregnancy outcomes, reported by two research teams, remains controversial (19, 20). Higher FOI was associated with higher implantation rate and live birth rate after first fresh embryo transfer (19). However, similar results were not found in another cohort of women of advanced age with unexplained infertility (20). To date, evidence on the relationship between FOI index and successful pregnancy in IVF is sparse and lacks systematization. Further investigations with large sample size are recommended to build conclusive evidence.

Therefore, by retrospectively analyzing 4,323 fresh embryo transfer cycles, the goal of our study is to investigate the relationship between the FOI and the clinical pregnancy rate (CPR) in IVF treatment.

## Materials and methods

### Study design

**Figure 1:**
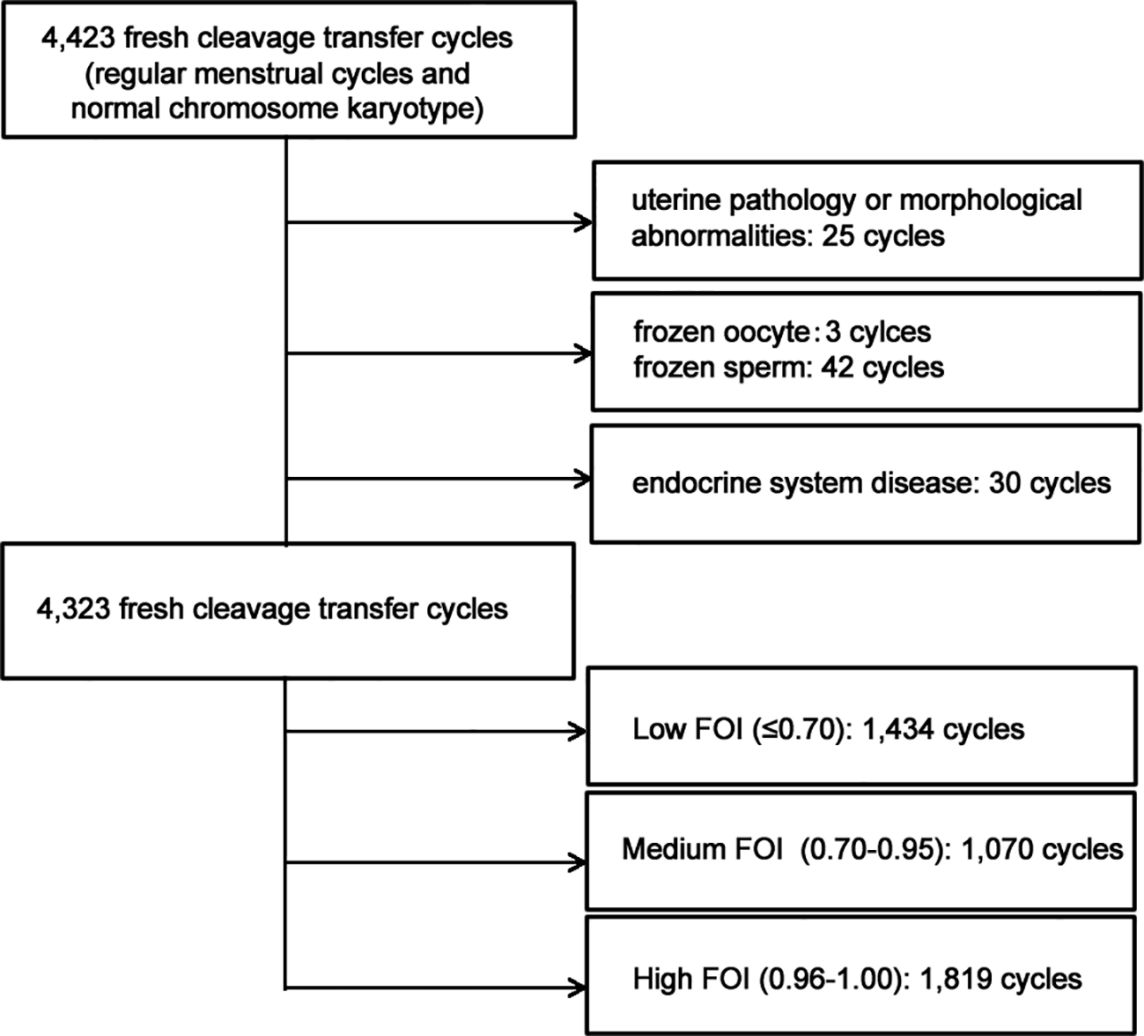
Flow chart of participant selection.

This retrospective study was performed in the Assisted Reproduction Center of Huizhou Central People’s Hospital, involving women who had undergone IVF treatment from 1 August 2011 to 31 January 2022. Ethical approval by the Ethics Committee of Huizhou Central People’s Hospital (number KYLL202203) was obtained. In light of the retrospective design, the requirement for informed consent was waived. The inclusion criteria were (1) fresh cleavage transfer cycles (2), regular menstrual cycles (the cycle length was approximately 25 to 35 days), and (3) women and their husbands with normal chromosome karyotype. The exclusion criteria were (1) uterine pathology or morphological abnormality (2), frozen oocyte or sperm, and (3) women who were diagnosed with any conditions affecting gonadal function or sex hormone secretion and excretion. **Figure 1** shows the flowchart of the study.

### Ovarian stimulation and oocyte retrieval

COH protocols were chosen according to maternal age, body mass index (BMI), AMH, basal hormone levels, and AFC. COH protocols consisted of long GnRH-agonist protocol and GnRH-antagonist protocol. Highly purified gonadotropin, either recombinant FSH or urinary FSH, was used. Women who underwent the long GnRH-agonist protocol were suppressed the function of the pituitary gland, by administration of a GnRH agonist (Diphereline^®^, Ipsen, France) in the midluteal phase of the previous cycle until human chorionic gonadotropin (hCG) day. As for the GnRH-antagonist protocol, Ganirelix (Orgalutran^®^, Organon) was given (0.25 mg/day s.c.) from day 5 of gonadotropin administration until hCG day, according to a fixed-dose regimen. The initial gonadotropin dose ranged from 150 to 375 IU/day, and subsequent adjustments were made according to the follicular growth during treatment. Follicular development was monitored by vaginal ultrasound every 2 or 3 days. While ≥2 follicles reached a diameter of ≥18 mm, patients were administrated 5,000–10,000 IU hCG, and oocyte retrieval was scheduled 34 to 36 h by vaginal ultrasound-guided aspiration.

### Embryo culture and scoring

According to the semen characteristics and previous fertilization history, routine IVF or intracytoplasmic sperm injection (ICSI) was performed. Rescue ICSI (RICSI) was performed if more than 60% of the MII oocytes did not show the second polar body after IVF insemination. During days 1 to 3 after insemination, embryos were cultured in pre-equilibrated cleavage medium K-SICM (Cook Medical Inc., Bloomington, IN, USA) with oil in multi-gas incubators (Minc, COOK, USA). According to the Istanbul consensus (21), fertilization check and embryo grading were performed at four time points (days 1, 3, 5, and 6). Fertilization was checked 16 to18 h after insemination/injection and considered normal fertilization if two pronuclei (2PN) were observed. Cleavage embryos (day 3) were evaluated on the basis of the scoring system by Gardner and Schoolcraft (22). A cleavage embryo was considered to be of good quality if it had seven to nine cells,<20% anucleate fragments, and no obvious morphological abnormality. Transcervical transfer of cleavage embryo was performed using a soft-tipped embryo transfer catheter. After embryo transfer, for luteal support, all patients received progesterone until 12 weeks after conception.

### Definition of FOI

FOI was defined as the ratio of the number of retrieved oocytes and AFC before COH. The AFC was evaluated as all identifiable antral follicles of 2–10 mm in diameter, as previously described (23). The FOI values were calculated using the following equation:


FOI = the number of retrieval oocytes ÷ AFC × 100.

### Definition of clinical pregnancy

The pregnancy outcomes were assessed according to the criteria published by the American Society for Reproductive Medicine in 2017 (24). Clinical pregnancy was defined according to the observation of at least one gestational sac by transvaginal ultrasonography and the detection of cardiac activity. CPR was expressed per embryo transfer.

### Data analysis and statistics

All parameters were collected from the electronic medical record system in hospital. The association of variables and CPR was first evaluated using univariate logistic regression analysis. Covariates were included as potential confounders if they changed the estimate of FOI on CPR by more than 10% or were thought to be clinically associated. Multivariable logistic regression analysis was conducted to assess the significance of independent variables. Association was calculated as crude odds ratio (OR) and adjusted ORs (aORs) with 95% confidence intervals (CIs). In addition, we evaluated the linearity of the association between FOI and CPR using a multivariate-adjusted generalized additive model (GAM) with splines. Subsequently, according to the covariates in the full model, interaction and stratified analyses were conducted. Covariates adjusted in the subgroup analyses were same as AdjustIImodel, except for the stratifying variables. *P*< 0.05 indicated a statistically significance. Data were analyzed using statistical software EmpowerStats (X&Y solutions Inc., USA) and R (http://www.R-project.org).

## Results

### Patient characteristics

In total, 4,323 fresh embryo transfer cycles were included (**Figure 1**). The baseline characteristics are listed in **Table 1**. The overall mean of FOI was 0.78 ± 0.24. When dividing FOI into tertiles, a higher FOI level was associated with younger female age, lower female BMI, and less previous failure cycle. No significant differences were found in male age, infertility duration, infertility type, and cause of infertility (**Table 1**).

**Table 1:** Baseline characteristics of patients.

FOI tertile	Total	Low	Medium	High	*P*-value
Number of patients	4,323	1,434	1,070	1,819	
Female age (years)	32.49 ± 4.65	32.75 ± 4.78	32.46 ± 4.59	32.30 ± 4.57	0.036
Male age (years)	34.71 ± 5.53	35.08 ± 5.74	34.56 ± 5.34	34.51 ± 5.46	0.050
Female BMI (kg/m^2^)	21.81 ± 3.18	22.15 ± 3.37	21.88 ± 3.18	21.49 ± 2.99	<0.001
Infertility duration (y)	4.08 ± 3.17	4.23 ± 3.32	4.08 ± 3.09	3.97 ± 3.10	0.124
Infertility type					
Primary	1,775 (41.06%)	584 (40.73%)	438 (40.93%)	753 (41.40%)	0.924
Secondary	2,548 (58.94%)	850 (59.27%)	632 (59.07%)	1,066 (58.60%)	
Cause of infertility					
Tubal factor	2,131 (49.29%)	708 (49.37%)	529 (49.44%)	894 (49.15%)	0.614
Endometriosis	145 (3.35%)	56 (3.91%)	30 (2.80%)	59 (3.24%)	
Unexplained	958 (22.16%)	327 (22.80%)	229 (21.40%)	402 (22.10%)	
Male factor	1,089 (25.19%)	343 (23.92%)	282 (26.36%)	464 (25.51%)	
AMH (ng/ml)	3.01 ± 1.22	3.03 ± 1.42	3.01 ± 1.22	2.98 ± 1.03	0.050
AFC (n)	13.19 ± 5.28	13.67 ± 6.58	12.87 ± 4.56	12.99 ± 4.40	0.321
Previous failure cycle	1.25 ± 0.64	1.32 ± 0.73	1.21 ± 0.58	1.22 ± 0.60	<0.001

Cutoff values of FOI tertiles: Tertile 1: ≤0.70; Tertile 2: >0.70, ≤0.95; Tertile 3: >0.96, ≤1.00.

### IVF/ICSI outcomes

The IVF/ICSI outcomes are presented in **Table 2**. The tertile 3 of FOI represented higher odds of utilizing agonist protocol, longer stimulation duration, more oocytes retrieved, and thicker endometrium. As to embryonic outcomes, more MII oocytes, more 2PN embryos, more useful embryos, and more better-quality embryos were observed in tertile 3. The tertile 3 of FOI had transferred with a greater number of embryo and better-quality embryo. Meanwhile, a higher CPR was shown in the higher FOI group. No significant groupwise differences were observed in fertilization type and 2PN embryo transferred (**Table 2**).

**Table 2:** IVF/ICSI outcomes of studied cycles stratified by FOI.

FOI tertile	Total	Low	Medium	High	*P*-value
COH protocol					
Agonist	3,532 (81.70%)	1,108 (77.27%)	868 (81.12%)	1,556 (85.54%)	<0.001
Antagonist	791 (18.30%)	326 (22.73%)	202 (18.88%)	263 (14.46%)	
Stimulation duration (days)	11.17 ± 2.04	11.17 ± 2.04	11.02 ± 1.92	11.34 ± 1.81	<0.001
No. of oocytes retrieved (n)	10.26 ± 4.86	6.57 ± 3.48	10.59 ± 3.89	12.99 ± 4.43	<0.001
Endometrial thickness (mm)	10.66 ± 2.07	10.53 ± 2.11	10.72 ± 2.01	10.73 ± 2.07	0.007
Triple-line endometrial pattern					
A	1,213 (28.06%)	458 (31.94%)	285 (26.64%)	470 (25.84%)	0.002
B	2,381 (55.08%)	746 (52.02%)	606 (56.64%)	1,029 (56.57%)	
C	729 (16.86%)	230 (16.04%)	179 (16.73%)	320 (17.59%)	
Fertilization type					
IVF	3,318 (76.75%)	1,109 (77.34%)	799 (74.67%)	1,410 (77.52%)	0.360
ICSI	840 (19.43%)	272 (18.97%)	222 (20.75%)	346 (19.02%)	
RICSI	165 (3.82%)	53 (3.70%)	49 (4.58%)	63 (3.46%)	
MII(n)	8.97 ± 4.50	5.86 ± 3.19	9.20 ± 3.79	11.29 ± 4.32	<0.001
2PN (n)	6.49 ± 3.68	4.30 ± 2.55	6.73 ± 3.30	8.08 ± 3.79	<0.001
Useful embryo (n)	4.13 ± 2.29	3.16 ± 1.72	4.26 ± 2.17	4.83 ± 2.49	<0.001
Good-quality embryo (n)	3.06 ± 2.42	2.13 ± 1.78	3.23 ± 2.40	3.69 ± 2.64	<0.001
No. of embryo transferred					
1	619 (14.32%)	271 (18.90%)	134 (12.52%)	214 (11.76%)	<0.001
2	3,582 (82.86%)	1,122 (78.24%)	909 (84.95%)	1,551 (85.27%)	
3	122 (2.82%)	41 (2.86%)	27 (2.52%)	54 (2.97%)	
2PN embryo transferred (%)	4,095 (94.73%)	1,343 (93.65%)	1,014 (94.77%)	1,738 (95.55%)	0.056
Good-quality embryo transferred					
All good-quality	3,267 (75.57%)	947 (66.04%)	840 (78.50%)	1,480 (81.36%)	<0.001
At least one good-quality	739 (17.09%)	328 (22.87%)	168 (15.70%)	243 (13.36%)	
All poor-quality	317 (7.33%)	159 (11.09%)	62 (5.79%)	96 (5.28%)	
Clinical pregnancy (%)	2,170 (50.20%)	678 (47.28%)	554 (51.78%)	938 (51.57%)	0.026

Cutoff values of FOI tertiles: Tertile 1: ≤0.70; Tertile 2: >0.70, ≤0.95; Tertile 3: >0.96, ≤1.00.

### Independent association between the FOI and the CPR

First univariate conditional logistic regressions were performed to analyze associations between collected variables and clinical pregnancy as dependent variable (**Supplemental Table 1**). We further took a multivariate logistic regression model (**Table 3**) to explore the independent association between the FOI and the CPR. After adjusted for confounders, FOI was significantly and positively associated with CPR (OR = 1.57; 95% CI: 1.18–2.11). Each standard deviation increments in FOI (SD = 0.24) corresponded to a 20% increase in the CPR. Grouped by dividing the total distribution into tertiles, a risk of 1.42 times was observed between the first and third groups after fully adjustment. A FOI cutoff value of 0.5 was used to categorize into high and low group. However, the CPR was not significantly higher in the high FOI group (**Supplemental Table 2**).

**Table 3:** Multivariate logistic regression of FOI for CPR.

Exposure	Non-adjusted	*P-*value	AdjustⅠ	*P*-value	AdjustⅡ	*P*-value
FOI	1.27 (1.09, 1.47)	0.0017	1.21 (1.04, 1.41)	0.0123	1.57 (1.18, 2.11)	0.0023
FOI (Per 1 SD increase)	1.10 (1.04, 1.17)	0.0017	1.08 (1.02, 1.15)	0.0123	1.20 (1.07, 1.35)	0.0023
Tertiles of FOI						
Tertile 1	Reference		Reference			
Tertile 2	1.20 (1.02, 1.40)	0.0261	1.18 (1.00, 1.38)	0.0474	1.30 (1.06, 1.58)	0.0099
Tertile 3	1.19 (1.03, 1.36)	0.0152	1.15 (1.00, 1.32)	0.0502	1.42 (1.09, 1.85)	0.0088
P for trend		0.0190		0.0360		0.010

AdjustImodel adjust for: female age and BMI.

AdjustIImodel adjust for: female age, female BMI, cause of infertility, infertility duration, previous failure cycle, protocol in fresh cycle, triple-line endometrial pattern, no. of MII oocyte, no. of useful embryo, no. of embryo transferred, 2PN embryo transferred, good-quality embryo transferred, AFC, and no. of oocytes retrieved.

Cutoff values of FOI tertiles: Tertile 1: ≤0.70; Tertile 2: >0.70, ≤0.95; Tertile 3: >0.96, ≤1.00.

### Linear association

**Figure 2:**
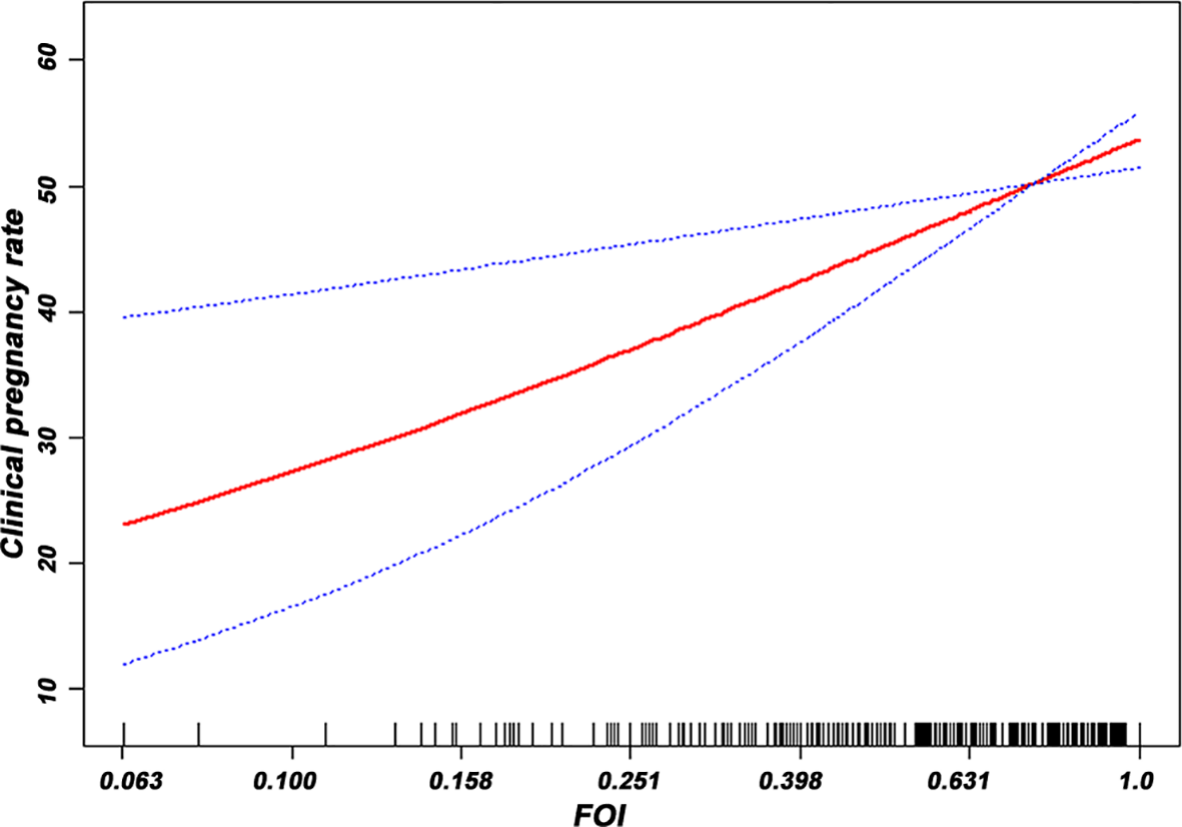
Smooth curve fitting was performed using GAM to assess the linearity of the association between the FOI and the CPR.

Smooth curve fitting was performed after adjusting for confounding factors in full model. The FOI-to-CPR relationship was shown to be linear within the entire range (**Figure 2**). This result agreed with the stepwise increased OR in the multivariate logistic regression analysis (**Table 3**; *P* for trend = 0.010 in the full model). However, there was no threshold effect within the range of FOI.

### Subgroup analysis

**Figure 3:**
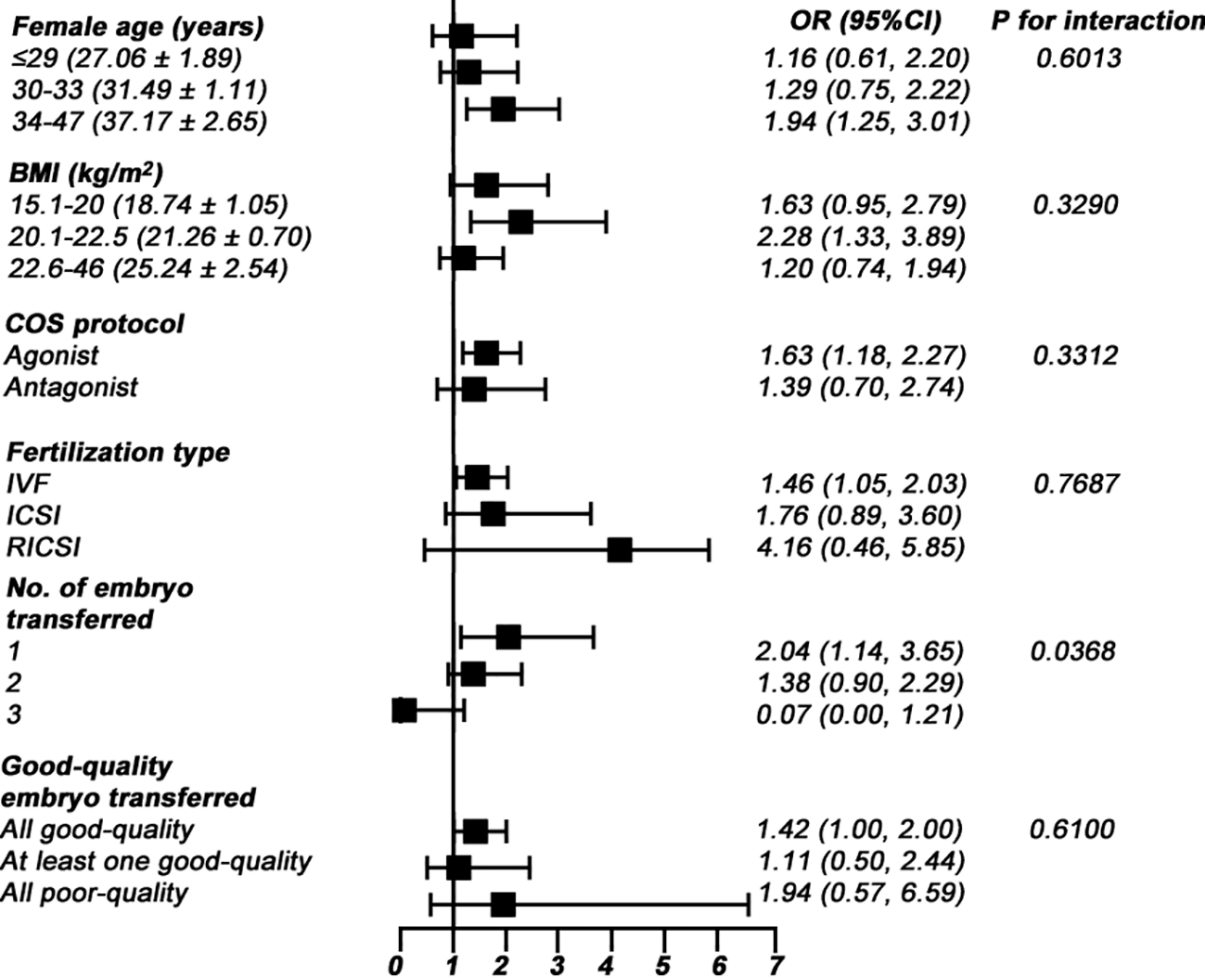
Subgroup analyses for the effect of FOI on CPR.

To find out whether the association between FOI and CPR was consistent across subgroups, we conducted subgroup analyses by numerous variables, as summarized in **Figure 3**. The results indicated that the association was consistent in subgroups of female age, BMI, COH protocol, fertilization type, and good-quality embryo transferred (**Figure 3**; all *P* for interaction > 0.05). We found that there was an interaction in the subgroup of the number of embryos transferred (**Figure 3**; *P* for interaction = 0.0368). The association between FOI and CPR was significantly stronger in the single cleavage transfer cycle (OR = 2.04; 95% CI: 1.14–3.65; *P* = 0.0156) (**Figure 3**).

## Discussion

In this large retrospective study, we found that FOI and CPR had a significant independent association in the fresh cleavage transfer cycle, after adjusted for reproductive related confounders. We further revealed that the relationship was linear over the entire range of FOI, but there was an absence of threshold effect. Furthermore, subgroup analyses implicated that the association was significantly stronger in the single cleavage transfer cycle. To the best of our knowledge, our research is the first study to report the independent linear association between FOI and CPR in IVF treatment, especially in single cleavage-stage embryo transfer cycle.

FOI is an index representing ovarian sensitivity to exogenous gonadotropin, which takes both retrieval oocytes and AFC into consideration. At the same time, FOI is an approach to indicate the ovarian resistance to gonadotropin stimulation. In general, low response to gonadotropin stimulation can be interpreted as the interaction effect between anthropometric characteristics, individual genotypes (25, 26), environmental contaminants (27), and oxidative stress (28). Because FOI may be the best to reflect ovarian response to COH, our study hypothesized that it can be used to predict pregnancy success in fresh embryo transfer cycle. The results verified our hypothesis. In the multivariate logistic regression models, we found an independently and positively linear correlation between FOI and CPR after adjusted for all possible variables. Specifically, each standard deviation increments in FOI (SD = 0.24) corresponded to a 20% increase in the CPR. Moreover, our result showed that a cutoff value above 0.5, as suggested in the previous literature (17), did not predict significantly higher CPR. Meanwhile, smooth curve fitting revealed that the prominent association was essentially linear across the entire range of FOI. Thus, FOI could be utilized as a linear marker to assess the likelihood to achieve clinical pregnancy in the fresh cleavage transfer cycle. Because no saturation or threshold effect was present in our analysis, future studies on the relationship between FOI and CPR should be cautiously interpreted.

To check the stability of the association between FOI and CPR in numerous subgroups, we carried out subgroup analyses and tests for interaction. The association remained robust in the subgroups of female age, BMI, COH protocol, fertilization type, and good-quality embryo transferred, suggesting that there was no synergistic or antagonistic effect between the FOI/CPR and these covariates. However, some subgroups deserved particular attention. Compared with women transferred with two or three cleavages, the OR value increased significantly in women who were transferred with single cleavage (OR = 2.04 vs. 1.38 vs. 0.07). Another observation that was worthy of further attention was that, in women transferred with two or three cleavages, the OR values were insignificant (*P* = 0.070 and 0.0673, respectively, not shown in **Figure 3**). Nevertheless, the sample sizes of these subgroups were 3,582 and 122 (82.86% and 2.82% of the total sampling, respectively), and both of the corresponding OR values were less than that in the total sampling. Moreover, we observed that the number of good-quality embryo was significantly higher in the higher FOI group (**Table 2**). This finding indicated that women with higher FOI had a sufficient number of good-quality embryos available for transfer that allowed for selection of the top-quality embryo. This effect may be most pronounced among women with elective single-embryo transfer but may be masked by double- or three-embryo transfer. In addition, the sample size in women transferred with three cleavages was too small to be sufficient statistical power to test the difference. Therefore, the clinical significance of the FOI/CPR association was more feasible to women who were transferred with single cleavage in fresh cycles. Nowadays, elective single-embryo transfer should be strongly promoted to reduce the obstetrical and perinatal risks of multiple pregnancies; therefore, our results have profound clinical implications.

To our knowledge, only a few studies in the literature regarded the predictive value of FOI in IVF treatment, and the results were inconsistent (19, 20, 29). A retrospective analysis included 264 IVF cycles and found that higher FOI was associated with the higher implantation rate and live birth rate after first fresh embryo transfer (19). These trends remained significant after adjusted for female age. Another literature reported that, in 740 IVF cycles, women with advanced age (≥39 years) and unexplained infertility, none of FOI, FORT, and OSI were not predictor of live birth, and the non-linear association existed between live birth and FOI (20). For FOI, their result seemed to be reasonably linear within the range of FOI from 0 to 1. However, a recent study retrospectively analyzed 298 IVF cycles with a cohort of women ≥39 years, and their findings suggested that FOI cannot predict live birth (20). In fact, the predictive value of the AFC was limited in older women, as they had mitochondrial dysfunction, increased apoptosis in granulosa cells, and increased oxidative stress in the follicular fluid (30, 31). Concerning FOI, analysis based on women with advanced age may lead to discrepant results.

Our study differs from these works in a few important ways. First, because of its definition, we assumed that FOI may exert a greater impact on fresh embryo transfer cycle. Our work chose CPR as the principal clinical outcome of IVF treatment and was first to found the existence of a linear relationship across the entire range of FOI. Second, our study collected a series of reproductive factors and only included covariates with a significant correlation. Previous literatures mentioned above were adjusted only for age and not corrected for other factors. Third, this is the first analysis to examine the interaction between covariates and implicated that the association between FOI and CPR might be more appropriate for women transferred with single cleavage in fresh cycles. Fourth, an important strength of our study is the large sample size.

There were limitations in our study. First, our study was retrospective design; therefore, residual confounding may have occurred due to unmeasured or unknown confounders, such as the reproductive factors of men. Second, a selection bias may be introduced by the retrospective design. Third, the variability in skill levels of operators during the oocyte retrieval procedure may also affect the total number of oocytes retrieved. However, our results were generated using actual clinical data from a single center, and the oocyte retrieval operations were performed by three senior physicians from our reproductive center during the study period. Thus, a large, multicenter, and prospective study may help overcome some of these limitations.

## Conclusions

Our study revealed a positive and linear association between FOI index and CPR in the fresh cleavage transfer cycle. Furthermore, we found that the association was significantly stronger in the single cleavage transfer cycle. As far as we know, our study, for the first time, evaluated the association between FOI index and clinical pregnancy outcome in Chinese population.

## Data availability statement

The original contributions presented in the study are included in the article/**Supplementary Material**. Further inquiries can be directed to the corresponding author.

## Ethics statement

The studies involving human participants were reviewed and approved by Ethics Committee of Huizhou Central People’s Hospital. The ethics committee waived the requirement of written informed consent for participation.

## Author contributions

PL and ZC contributed to the study conception and design. ZC participated in data analysis and prepared the figure and tables. PL wrote the manuscript text. All authors reviewed and approved the final manuscript.

## Acknowledgments

The authors express their gratitude to the staff from Huizhou Central People’s Hospital for collecting the data and to all the participants for their assistance.

## Conflict of interest

The authors declare that the research was conducted in the absence of any commercial or financial relationships that could be construed as a potential conflict of interest.

## Publisher’s note

All claims expressed in this article are solely those of the authors and do not necessarily represent those of their affiliated organizations, or those of the publisher, the editors and the reviewers. Any product that may be evaluated in this article, or claim that may be made by its manufacturer, is not guaranteed or endorsed by the publisher.

## Abbreviations

AMH, anti-Müllerian hormone; AFC, antral follicle count; BMI, body mass index; CI, confidence interval; COH, controlled ovarian hyperstimulation; CPR, clinical pregnancy rate; FOI, follicle-to-oocyte index; FORT, follicular output rate; FSH, follicle-stimulating hormone; GAM, generalized additive model; hCG, human chorionic gonadotropin; ICSI, intracytoplasmic sperm injection; IVF, *in vitro* fertilization; OR, odds ratio; OSI, ovarian sensitivity index; 2PN, two pronuclei.
