# Supplementary material for: Association of follicle-to-oocyte index and clinical pregnancy in IVF treatment: A retrospective study of 4,323 fresh embryo transfer cycles
Source: Front Endocrinol (Lausanne). 2022 Oct 3;13:973544. doi: 10.3389/fendo.2022.973544 (PMC9574222; doi:10.3389/fendo.2022.973544)
Supplement: Supplementary file 1 [file Table_1.docx]

Supplemental table 1. Univariate analysis.

| Characteristics |  | *P* value |
| --- | --- | --- |
| Female age (y) | 0.93 (0.91, 0.94) | <0.0001 |
| Male age (y) | 0.95 (0.94, 0.96) | <0.0001 |
| Female BMI (kg/m2) | 0.98 (0.96, 1.00) | 0.0584 |
| Infertility duration (y) | 0.96 (0.94, 0.97) | <0.0001 |
| Infertility type |  |  |
| Primary | 1.0 |  |
| Secondary | 0.84 (0.75, 0.95) | 0.0054 |
| Cause of infertility |  |  |
| Tubal factor | 1.0 |  |
| Endometriosis | 0.77 (0.55, 1.08) | 0.1310 |
| Unexplained | 0.90 (0.78, 1.05) | 0.1965 |
| Male factor | 1.06 (0.91, 1.22) | 0.4715 |
| AMH (ng/ml) | 1.08 (1.03, 1.14) | 0.0016 |
| AFC (n) | 1.01 (1.00, 1.03) | 0.0452 |
| Previous failure cycle | 0.90 (0.81, 0.99) | 0.0337 |
| Protocol in fresh cycle |  |  |
| Agonist | 1.0 |  |
| Antagonist | 0.68 (0.59, 0.80) | <0.0001 |
| Stimulation duration (days) | 1.04 (1.01, 1.07) | 0.0137 |
| Endometrial thickness (mm) | 1.12 (1.09, 1.16) | <0.0001 |
| Triple-line endometrial pattern |  |  |
| A | 1.0 |  |
| B | 1.45 (1.25, 1.67) | <0.0001 |
| C | 1.89 (1.56, 2.28) | <0.0001 |
| No. of oocytes retrieved (n) | 1.04 (1.02, 1.05) | <0.0001 |
| IVF/ICSI |  |  |
| IVF | 1.0 |  |
| ICSI | 0.93 (0.80, 1.08) | 0.3294 |
| RICSI | 0.87 (0.64, 1.19) | 0.3914 |
| MII (%) | 1.32 (0.91, 1.92) | 0.1491 |
| 2PN (%) | 1.38 (1.05, 1.80) | 0.0208 |
| Useful embryo (%) | 1.43 (1.08, 1.90) | 0.0116 |
| Good-quality embryo (%) | 2.52 (1.90, 3.35) | <0.0001 |
| No. of embryo transferred |  |  |
| 1 |  |  |
| 2 | 1.65 (1.38, 1.96) | <0.0001 |
| 3 | 1.42 (0.96, 2.10) | 0.0779 |
| 2PN embryo transferred (%) | 0.63 (0.48, 0.83) | 0.0010 |
| Good-quality embryo transferred (%) |  |  |
| All good-quality | 1.0 |  |
| At least one good-quality | 0.67 (0.57, 0.79) | <0.0001 |
| All poor-quality | 0.45 (0.35, 0.57) | <0.0001 |
| Previous failure cycle | 0.90 (0.81, 0.99) | 0.0337 |
| FOI | 1.44 (1.12, 1.84) | 0.0042 |
