# Supplementary material for: Association of follicle-to-oocyte index and clinical pregnancy in IVF treatment: A retrospective study of 4,323 fresh embryo transfer cycles
Source: Front Endocrinol (Lausanne). 2022 Oct 3;13:973544. doi: 10.3389/fendo.2022.973544 (PMC9574222; doi:10.3389/fendo.2022.973544)
Supplement: Supplementary file 2 [file Table_2.docx]

Supplemental table 2. Multivariate logistic regression of FOI groups for CPR.

| Exposure | Non-adjusted^a^ | *P* value | Adjust I^b^ | *P* value | Adjust II^c^ | *P* value |
| --- | --- | --- | --- | --- | --- | --- |
| FOI group |  |  |  |  |  |  |
| <0.5 | Reference |  | Reference |  | Reference |  |
| ≥0.5 | 1.22 (1.03, 1.45) | 0.0227 | 1.17 (0.98, 1.39) | 0.0748 | 1.20 (0.95, 1.50) | 0.1226 |

AdjustⅠmodel adjust for: female age and BMI.

AdjustⅡmodel adjust for: female age, female BMI, cause of infertility, infertility duration, previous failure cycle, protocol in fresh cycle, triple-line endometrial pattern, no. of MII oocyte, no. of useful embryo, no. of embryo transferred, 2PN embryo transferred, good-quality embryo transferred, AFC and no. of oocytes retrieved.
